# Depletion of White Adipose Tissue in Cancer Cachexia Syndrome Is Associated with Inflammatory Signaling and Disrupted Circadian Regulation

**DOI:** 10.1371/journal.pone.0092966

**Published:** 2014-03-25

**Authors:** Maria Tsoli, Martina Schweiger, Anne S. Vanniasinghe, Arran Painter, Rudolf Zechner, Stephen Clarke, Graham Robertson

**Affiliations:** 1 Experimental Therapeutics, Children’s Cancer Institute for Medical Research, Randwick, NSW, Australia; 2 Institute of Molecular Biosciences, University of Graz, Graz, Austria; 3 Cancer Pharmacology Unit, Anzac Research Institute, Concord Repatriation General Hospital, Concord West, NSW, Australia; 4 Northern Clinical School, University of Sydney, Royal North Shore Hospital, St Leonards, NSW, Australia; 5 Tumorkine Research, Garvan Institute of Medical Research, Darlinghurst, NSW, Australia; University of Warwick – Medical School, United Kingdom

## Abstract

Involuntary weight loss in patients with cancer is the hallmark of cancer cachexia. The etiology of cachexia is multifactorial involving loss of skeletal muscle and adipose tissue associated with high systemic levels of acute phase proteins and inflammatory cytokines. While muscle wasting overtly impacts on cancer patient quality of life, loss of lipid depots represents a sustained energy imbalance. In this study fat depletion was examined in Colon-26 model of cancer cachexia, which is a widely used rodent model of this syndrome. We investigated diurnal expression of circadian rhythm regulators as well as key mediators of energy metabolism and cytokine signaling. Mice bearing the C26 tumour exhibited reduced adipose mass, elevated adipose tissue lipolysis and a 5-fold increase in plasma levels of free fatty acids. These changes were associated with activated IL-6 signaling in WAT through a 3-fold increase in phosphorylated STAT3 and high SOCS3 gene expression levels. In addition perturbations in circadian regulation of lipid metabolism were also observed. Lipid catabolism did not appear to be influenced by the classical PKA pathway activating the lipase HSL. ATGL protein levels were elevated 2-fold in cachectic mice while 4-fold increase phosphorylated ACC and a 2-fold decrease in phosphorylated 4EBP1 was observed indicating that lipid metabolism is modulated by the ATGL & AMPK/mTOR pathways. This study provides evidence for activation of cytokine signaling and concomitant alterations in circadian rhythm and regulators of lipid metabolism in WAT of cachectic animals.

## Introduction

Cancer cachexia syndrome (CCS) is frequently experienced by advanced cancer patients being most prevalent in pancreatic, upper gastrointestinal and lung cancers and accounts for nearly 20% of cancer-related morbidities [1], [2]. Currently there are no effective treatments or prognostic tests to indicate those patients at risk of developing cachexia. CCS is a complex metabolic disorder characterized by involuntary weight loss, adipose tissue and skeletal muscle depletion, anorexia and fatigue. Cachexia is often associated with systemic inflammation indicated by elevated plasma CRP and reduced albumin levels [3]. In addition cachectic cancer patients often experience increased toxicity during chemo/radiotherapy leading to reduced quality of life and subsequent poor survival [4], [5]. Although the exact mechanism is yet to be fully elucidated, it is widely accepted that increased levels of cytokines such as interleukin-6 (IL-6), tumour necrosis factor (TNF) and other factors such as zinc-alpha2-glycoprotein (ZAG) trigger the catabolic events that promote lipid depletion from fat depots and asthenia [6], [7], [8]. In particular, IL-6 has been implicated in the development of cachexia in tumour-bearing rodent models and loss of adipose tissue in cancer patients [9], [10] as well as directly stimulating lipolysis in adults [11]. The Colon-26 (C26) carcinoma is a well-established murine model of cancer cachexia resulting in high systemic levels of IL-6. Administration of agents that inhibit IL-6 signaling prevent wasting and other features of cachexia in several animal models of cachexia including Colon 26 [12], [13], [14].

In addition to acting as an insulator and lipid reservoir during periods of metabolic demand, the role of white adipose tissue (WAT) extends to neuroendocrine control of energy homeostasis, appetite and immune/inflammatory responses. While mobilization of lipids from adipocytes to supply energy to other organs during fasting/calorie restriction is the primary function of WAT, it is also involved in the secretion of adipokines and cytokines, with many adipokines exhibiting diurnal variations in plasma which can influence appetite, energy expenditure and reproduction [15]. Conversely distal organs control lipolytic vs lipogenic pathways in WAT via hormones and cytokines to regulate lipid metabolic pathways and thereby achieve whole body energy balance. Therefore loss of WAT mass during the development of cachexia would impact on multiple organ systems beyond impaired provision of energy rich lipids.

The secretion and activity of diurnal regulators of metabolism are linked to the body’s own endogenous clock to match nutrient supply with activity cycles and environmental cues [16]. Central control of circadian rhythm is mediated by the suprachiasmatic nucleus in which an auto-regulated molecular feedback mechanism involving the BMAL/Clock regulators and Per/Cry target proteins operates to exert neural regulation on clock functions in peripheral organs. An additional feedback loop involves the nuclear receptor Rev-erbα, which also plays an essential role in adipogenesis. Molecular analysis of WAT found that ∼20% of the adipose transcriptome exhibits a rhythmic diurnal expression pattern [17]. In fact many nuclear transcription factors such as the peroxisome proliferator-activated receptors (PPARs), as well as the key energy homeostatic regulator AMP activated protein kinase (AMPK) and metabolic enzymes (lipoprotein lipase-LPL) exhibit oscillatory profiles indicating complex interactions between circadian clock, energy balance and environmental cues [18]. Circadian dysregulation is associated with higher risk of metabolic syndrome comprising obesity, insulin resistance and cardiovascular disease [19], [20]. Mouse models of genetically manipulated circadian clock components Bmal or Clock display features of metabolic syndrome-like phenotypes or leanness respectively [21], [22]. Furthermore, clinical studies have demonstrated increased risk of obesity and its comorbidities in night-shift and jet-lagged workers [20], [23].

Previous studies found that white adipose tissue is one of the first organs to be affected during cancer cachexia with WAT depletion often evident in the absence of overt lean body mass loss [9]. Loss of adipose tissue is mediated predominantly by increased lipolysis associated with a hypermetabolic state and to some extent reduced fat deposition [24], [25], [26]. The mobilization of lipid reserves in adipose tissue during cachexia is mediated by adipose tissue triglyceride lipase (ATGL) and hormone sensitive lipase (HSL) [24]. The critical role that ATGL plays in cancer cachexia was highlighted by not only the preservation of fat mass, but also the lack of skeletal muscle wasting, in ATGL deficient mice with cachexia-inducing Lewis Lung carcinomas or B16 melanomas [24]. While these studies extend our understanding of the contribution these lipases make to adipose tissue integrity in cancer as well as other metabolic states [27], the signaling mechanism that initiates fat depletion in cachexia remains poorly understood [28], [29]. Several questions remain unanswered in regards to the hypermetabolic features - especially increased lipolysis - apparent in cancer cachexia. What are the links between enhanced lipolysis and systemic inflammation associated with cancer cachexia? Is increased cytokine signaling operative during adipose tissue depletion? How is the diurnal expression of master regulators of circadian rhythm and lipid metabolism affected during cancer cachexia? Are the normal diurnal levels of key sensors and mediators of nutritional state and lipid metabolism altered in WAT?

In this study we investigated the impact of cachectic colon-26 carcinoma on white adipocyte diurnal rhythmicity and function. This murine model of cancer cachexia has been widely used to study cachexia as it exhibits similar functional and metabolic impairments to the clinical condition [30]. We specifically characterized the diurnal expression of genes important for circadian rhythm control and lipid metabolism as well as the activation state of key regulators of energy homeostasis at two opposite time points in the diurnal cycle. We show evidence for cytokine signaling through components of the IL-6 signaling cascade - STAT3 and SOCS3. In addition, we report that stimulation of the lipolytic pathway in WAT of cachectic mice is associated with increased ATGL and perilipin rather than via PKA/HSL. Our data identifies potential cross-talk between AMPK, mTOR and 4EBP1 as important mediators of altered lipid metabolism in cancer cachexia.

## Materials and Methods

### Cell Culture and Animal Studies

The cachexia-inducing Colon 26 cells (C26) is a murine colon adenocarcinoma and were kindly provided as a gift by AMGEN (USA) [31]. C26 cells were grown and maintained in RPMI medium (Invitrogen) containing 10% fetal bovine serum (Invitrogen) and 100 μg/mL Penicillin/Streptomycin (Invitrogen) in a 5% CO_2_ environment. Pathogen-free, 10–12 week-old male BALB/c*DBA2 (F1 Hybrid) mice were purchased from ARC, Perth (Australia), and kept at an ambient temperature of 22°C under a 12 hour light cycle (lights on 6∶00 am to 6∶00 pm). Colon 26 cells were inoculated subcutaneously at 1×10^6^ cells/100 μL into the right flank of mice. Control mice were injected with 100 μL of RPMI medium containing antibiotics. Over a period of 14 days following inoculation, body weight, food intake and tumor dimensions were recorded daily. Tumor-bearing and free-fed control mice had *ad libitum* access to food. One group of control mice was pair-fed to match the daily food intake of cachectic C26-bearing group. Pair-feeding was achieved by giving non-tumor bearing control mice the food intake of corresponding C26-tumour bearing animals. Mice were euthanazed by cervical dislocation before harvesting epididymal adipose tissues that were snap frozen in liquid nitrogen. Animal experimentation was performed according to the Australian Code of Practice for the Care and Use of Animals for Scientific Purposes under the Animal Research Regulation (2005) of NSW. The impact of tumour growth and development of cachexia on animal welfare were considered, with appropriate measures to monitor and alleviate suffering implemented, under protocol #2007/006 approved by the Animal Welfare Committee of Sydney South-West Area Health Service.

### Evaluation of Cachexia and Biochemical Analysis

Fourteen days following tumor cell inoculation, the mice were anaesthetized with ketamine/xylazine and euthanazed after the blood collection by cardiac puncture. The net weight of the carcasses was measured. Epididymal fat was collected, weighed and then stored at −80°C until use. Aliquots of plasma obtained from the blood of each mouse were kept frozen until further analysis. The non-esterified fatty acid content in the plasma of cachectic animals was determined enzymatically using the NEFA C kit (Wako Pure Chemicals/Novachem, Collingwood, VIC, Australia). The triglyceride content in the plasma of cachexia inducing C26-bearing animals was determined using the clinical automater analyser (Roche Modular E170) following the manufacturer’s instructions (Roche).

### Adipose Tissue Histology and Microscopy

WAT samples were fixed in 10% formalin neutral buffered solution (Sigma-Aldrich), embedded in paraffin wax, and 5 μm sections cut and mounted on glass slides. After dehydration, the sections were stained with hematoxylin-eosin (H/E) for histological examination by light microscopy and CAST grid software (Olympus Corp., Albertslund, Denmark). Morphological analysis was performed tissue obtained from 4 animals per group (control, C26-bearing mice and pair-fed). Surface area and perimeter were estimated from approximately 5–8 different fields of view per image giving a total of giving 250 per animal i.e 1000 adipocytes per experimental group.

### Lipolysis of Isolated WAT Organ Cultures

Gonadal fat pads were surgically removed and washed several times with PBS. Tissue pieces (∼15 mg) were pre-incubated for 2 h in DMEM in the presence or in the absence of 25 μM Hi-76-0079 (Novo Nordisk) at C37°C, 5% CO_2,_ 95% humidified atmosphere. Thereafter, the medium was replaced by DMEM containing 2% BSA (fatty acid-free) either in the presence or in the absence of 10 μM forskolin and 25 μM Hi-76-0079 and incubated for another 60 min at 37°C. Aliquots of the medium were removed and analyzed for FFA and glycerol content using commercial kits (HR Series NEFA-HR(2), WAKO Diagnostics). For protein determinations, fat pads were washed extensively with PBS and lysed in 0.3 N NaOH/0.1% SDS. Protein measurements were performed using the BCA reagent (Pierce).

### Quantitative Real-Time PCR Analysis

Total RNA was isolated using Trizol (Invitrogen) according to the manufacturer’s protocol. First-strand cDNA was synthesized from total RNA using SuperScript III with random primers (Invitrogen) and oligo(dT)12-14 (Invitrogen). All primer sequences were BLASTed against the NCBI mouse genomic sequence database to ensure specificity for the corresponding gene. Primer sequences are provided in Table S1. qPCR reactions contained 1.25 ng cDNA, 300 nM gene-specific primers and 12 μL SybrGreen (Invitrogen). All reactions were performed using the Corbett Rotor Gene 6000 (Corbett Life Science). Relative mRNA levels were calculated by the comparative threshold cycle method by using *36B4*, *Tfrc* and *Hmbs* genes as the housekeeping controls.

### Western Blotting

Whole-cell extracts from adipose tissues were obtained using RIPA Lysis Buffer supplemented with phosphatase and protease inhibitors (Roche) using a glass homogenizer and concentrated with centrifugal filter units according to manufacturer’s protocols (Cell Signaling Technologies, Millipore) and were subsequently quantified by BCA protein acid assay kit (Pierce). Proteins (20 μg) were resolved on 10% Tris-HCl gels (Bio-Rad), and blotted (iBlot, Invitrogen) as described by manufacturers. Cell lysates were analyzed with the following primary antibodies: rabbit anti–phospho-p44/42-mitogen-activated protein kinase (MAPK)-Thr202/Tyr204, rabbit anti-p44/42-MAPK, rabbit anti-phospho-p38 MAPK-Thr180/Tyr182, rabbit anti-p38 MAPK, rabbit anti-signal transducer and activator of transcription 3 (STAT3), rabbit anti-phospho-STAT3-Ser727, rabbit anti-ATGL rabbit anti-hormone-sensitive- lipase (HSL), rabbit anti-phospho-HSL-Ser563, rabbit anti-acetyl-coA-carboxylase (ACC), rabbit anti-perilipin, rabbit anti-AMP-activated protein kinase α (AMPKα), rabbit anti-phospho-AMPKα-Thr172, rabbit anti-raptor, rabbit anti-mammalian target of rapamycin (mTOR), rabbit anti-phospho-mTOR-Ser2481, rabbit anti-eukaryotic translation initiation factor 4E-binding protein 1 (4EBP1), rabbit anti-phospho-4EBP1-Thr37/46, rabbit anti-phospho-4EBP1-Thr70, rabbit anti-phospho-4EBP1-Ser65, rabbit anti-3-Hydroxyacyl CoA dehydrogenase (EHHADH/PBE) and rabbit anti-beta actin. All anti-phospho primary antibodies were diluted 1∶500 while the remaining antibodies were diluted 1∶1000. Proteins were visualized using the goat anti-rabbit-HRP secondary antibody (1∶2000) and west-femto kit (Pierce). Apart from EHHADH (Abcam) the rest of antibodies were obtained from Cell Signaling Technology.

### Statistical Analysis

Data are presented as mean±s.e.m. Statistical analyses were carried out with unpaired Student’s t-tests. Analysis between 3 groups (control, colon-26 and pair-fed) was performed using the One-way Anova Post Hoc Turkey test. Differences were considered statistically significant at P<0.05.

## Results

### Impact of C26 Carcinoma on Epidydimal White Adipose Tissue

**Figure 1:**
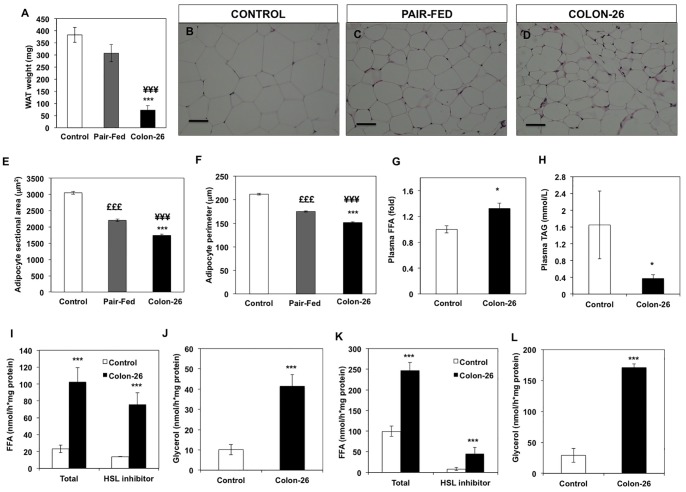
Organ weight and morphological characteristics of white adipose tissue from cachectic C26 tumour-bearing, *ad lib* and pair-fed non-tumor bearing control mice. (A) Epididymal adipose tissue weights from cachectic animals at day 14 after tumour inoculation (^***^P<0.001, ^¥¥¥^P<0.001 vs pair-fed); (C–E) Hematoxylin and eosin staining and (B–F) quantification of adipocyte size from cachectic and pair-fed mice. (G) Circulating levels of non-esterified free fatty acids in plasma of cachectic animals (*P<0.05; vs control); (H, I) Free fatty acid (FFA) and glycerol release from WAT explants obtained from control and C26 tumour-bearing mice under basal conditions in the presence and in the absence of the HSL inhibitor Hi 76–0079; (J, K) Free fatty acid (FFA) and glycerol release from WAT explants under forskolin stimulated conditions in the presence or in the absence of Hi- 76–0079. For A, values are presented as mean ± s.e.m. for 8–10 animals per group. For B, C and D representative images are shown from H&E images taken from 4 animals per group. For E and F values are presented as as mean ± s.e.m. for 4 animals per group. Approximately 250 adipocytes were counted per animal giving in total 1000 adipocytes from each group. G and H values are presented as mean ± s.e.m. for 4 animals per group. For I–K values are presented as mean ± s.e.m. for 5 animals per group.

The Colon26 carcinoma is a well-established model of cancer cachexia in mice exhibiting significant weight loss, muscle wasting, fat depletion and gradual decrease of food intake 14 days after tumour implant. In agreement with previous reports, the mass of epididymal WAT was considerably reduced compared to free-fed and pair-fed controls (Figure 1A). We examined whether this dramatic reduction in adipose tissue mass was apparent at the microscopic level. Haematoxylin/Eosin staining showed significant morphological alterations. White adipocytes from control mice displayed single hexagonal-shaped lipid vacuoles that were smaller in pair-fed animals and appeared more spherical in cachectic mice (Figure 1B–D). Morphometric analysis further showed a significant decrease in sectional area and perimeter of white adipocytes from C26 cachectic mice compared with *ad lib* and pair-fed non-tumour bearing control mice (Figure 1E–1F). The reduction in WAT weight was accompanied by an increase in plasma non-esterified fatty acids (NEFA) in cachectic mice (Figure 1G) and a decrease of plasma triglyceride levels (Figure 1H). To examine the contribution of lipases to the mobilization of triglycerides from adipose tissue, we determined the lipid mobilization activity of WAT explants. The rate of free fatty acid (FFA) release was 5-fold higher in WAT of C26 cachectic mice compared to control non-tumor bearing mice (Figure 1I) with a corresponding increase in glycerol release (Figure 1J). The addition of the HSL-specific inhibitor (Hi 76–0079) only marginally reduced FFA release indicating that ATGL is the predominant lipase responsible for adipose tissue lipolysis (Figure 1I). To assess the impact of cachexia on the total lipid hydrolytic capacity after hormonal stimulation, WAT tissue was treated with forskolin. A further increase in FFA release was apparent for both control and C26 cachectic mice, with cachectic mice attaining a maximal rate of FFA production of 250 nmol/hr*mg protein compared with 100 nmol/hr*mg in control mice (Fig. 1K). Glycerol release also showed a higher maximal rate in C26 versus control mice after stimulation of WAT explants with forskolin (Figure 1L) indicating a higher lipolytic capacity of WAT explants from C26 cachectic mice.

### Activation of IL-6 Signaling Pathway in C26 Cachexia

**Figure 2:**
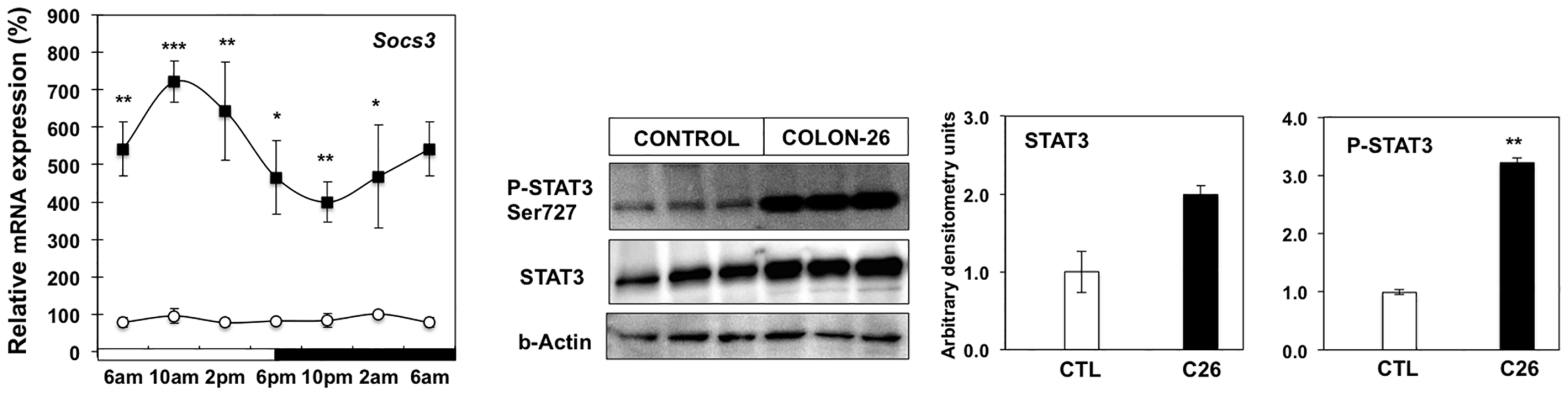
Activation of IL-6 signaling pathway in C26-bearing mice. Diurnal mRNA expression of SOCS3 mRNA in white adipose tissue from control mice (white circle) and cachectic mice (black square) (^*^P<0.05, ^**^P<0.01, ^***^P<0.001 vs control). 6 am values are duplicated in the graph to illustrate diurnal rhythmicity. mRNA expression values are mean ± s.e.m. relative to controls for 4–6 animals per group. Western blot and densitometric analysis of STAT3 protein and phospho-STAT3 (Ser727), in white adipose tissue from cachectic and control animals.

Cytokines such as IL-6 appear to be key drivers of the wasting process in this cancer cachexia model [12]. To determine whether active cytokine signaling is present in the adipocytes from cachectic mice we investigated the diurnal mRNA expression of SOCS3, and phosphorylation of STAT3 (Figure 2). Transcript levels of SOCS3 mRNA were significantly increased at all time points in cachectic mice. There was also enhanced phosphorylation of STAT3 protein (Ser727) in the presence of the C26 tumor. These data demonstrate active signaling downstream of the IL-6 receptor in WAT of cachectic mice that may influence lipid metabolic pathways in cachectic mice.

### Effect of Cancer Cachexia on Diurnal Expression Pattern of Lipogenesis Pathway

**Figure 3:**
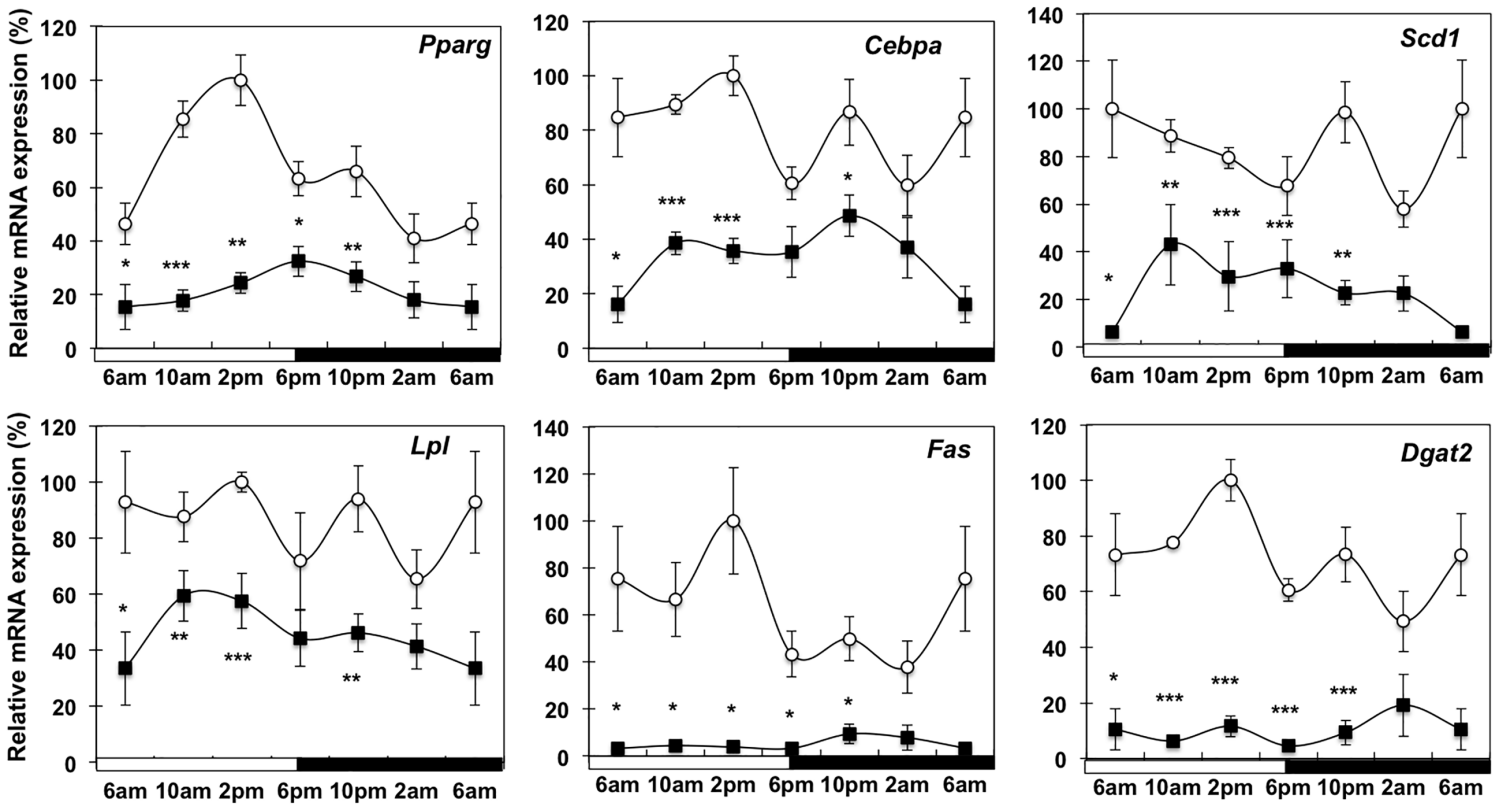
Expression of genes involved in lipid biosynthesis pathway in white adipose tissue during cancer cachexia. mRNA analysis of *Pparγ*, *C/ebpα*, *Lpl*, *Fas*, *Scd1*, *Dgat2*, isolated from WAT of control (white circle), and C26-bearing mice (black square); Values are mean ± s.e.m. presented as percentages relative to the controls for 4–5 animals per group (*P<0.05; **P<0.01; ***P<0.001 C26 vs control). 6 am values are duplicated in each graph to illustrate a complete 24-h cycle.

To examine the impact of cancer cachexia on *de novo* synthesis of fatty acids in WAT we investigated the diurnal expression profile of metabolic genes and nuclear transcription factors involved in lipogenesis. Lipoprotein lipase (LPL) is a key enzyme involved in TG hydrolysis of lipoproteins to release fatty acids for uptake into cells. In control mice its transcript levels exhibited no diurnal rhythm while in cachectic mice expression levels were significantly dampened at most time points (Figure 3). Together with *Lpl*, the expression of *Ap2* was reduced while *Cd36* was not altered in WAT of cachectic mice indicating some degree of repression in uptake or intracellular handling of fatty acids (Figure S1). PPAR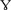 is the predominant nuclear transcription factor regulating lipid metabolism in adipocytes. In control mice, *Ppar* 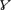 and associated target genes *Fas* and *Dgat2* peaked during the light cycle, while in the tumor-bearing cachectic animals rhythmicity was lost and mRNA levels were markedly decreased at most time points. Similarly *Scd1* exhibited reduced mRNA expression in cachectic mice. C/EBPα is important for adipogenesis and normal adipocyte function. In control mice *C/ebpα* did not exhibit a diurnal expression pattern while in cachectic mice transcript levels were significantly reduced throughout the day. Interestingly direct comparisons at the gene expression levels for *Fas* and *C/ebpα* between pair-fed mice and cachectic mice indicated significant differences at 2 am time point indicating that lipid synthesis might be mainly affected during cachexia and not as much during caloric restriction.

### PKA-induced Lipolytic Pathway is not Enhanced in Cancer Cachexia

**Figure 4:**
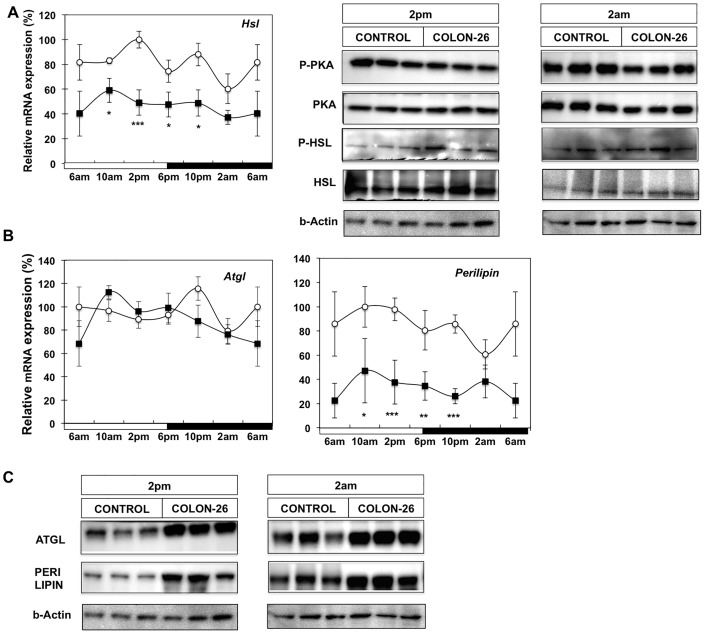
Lipolytic pathway in WAT of cachectic animals. (A) mRNA analysis of *Hsl* gene expression and Western blot analysis of phospho-PKA (Thr197), total PKA, phospho-HSL and total HSL proteins at 2 am and 2 pm; (B) mRNA analysis of *Atgl* and *Perilipin* gene expression from WAT of control (white circle), and C26 tumour-bearing mice (black square); Values are mean ± s.e.m. presented as percentages relative to the controls for 4–5 animals per group (*P<0.05; **P<0.01; ***P<0.001, C26 vs control). 6 am values are duplicated in each graph to illustrate a complete 24-h cycle; (C) Western blot analysis of ATGL and PERILIPIN proteins at 2 pm and 2 am.

Stimulation of FFA release from triglyceride was thought to be primarily mediated via protein kinase A (PKA), which directly phosphorylates perilipin and hormone sensitive lipase (HSL) and also influences adipose triglyceride lipase (ATGL), albeit indirectly. However recent studies have identified AMPK-mediated phosphorylation of ATGL and interactions with CGI-58 as pivotal for maximal ATGL hydrolase activity [27]. We assessed whether lipolysis is mediated through the PKA pathway during depletion of WAT in cancer cachexia. As shown in figure 4A, we observed a slight decrease in phosphorylated PKA and total PKA levels at 2 am, while at 2 pm no apparent changes were noted between control and cachectic animals (Figure S2). mRNA expression levels appeared reduced while total and phospho-HSL protein levels were unchanged at either time-point during cancer cachexia. Interestingly at gene expression level no significant changes were observed between control and pair-fed mice (Figure S3), whereas at the protein level, we observed increased phosphorylation in both PKA and HSL at 2 am (Figure S3). Perilipin is a pivotal lipid coating protein for fat droplet formation and is directly influenced by PKA. We investigated whether cancer cachexia influenced the mRNA and protein levels of perilipin and found no apparent diurnal rhythm in the transcript levels of perilipin in control mice, while expression was reduced in cachectic animals (Figure 4B). However Western analysis showed increased levels of perilipin protein at both time-points (Figure 4C, Figure S2). ATGL is the critical lipase that initiates the first step in triglyceride degradation. We did not observe circadian rhythmicity in expression of this gene and there was no impact on ATGL mRNA during cancer cachexia. However, Western analysis revealed increased ATGL protein level for both time-points in WAT from C26 tumour-bearing animals (Figure 4, Figure S2). Similar to the apparent discordance in perilipin protein and mRNA levels, it appears that ATGL is predominantly regulated at a post-transcriptional level in cachexia. These data indicate that during late stage cachexia, fat mobilization from WAT of C26 mice is not induced by the PKA pathway to stimulate HSL-mediated lipolysis, but is associated with increased ATGL protein.

#### Perturbed diurnal expression of fatty acid utilization pathway in cachectic mice

**Figure 5:**
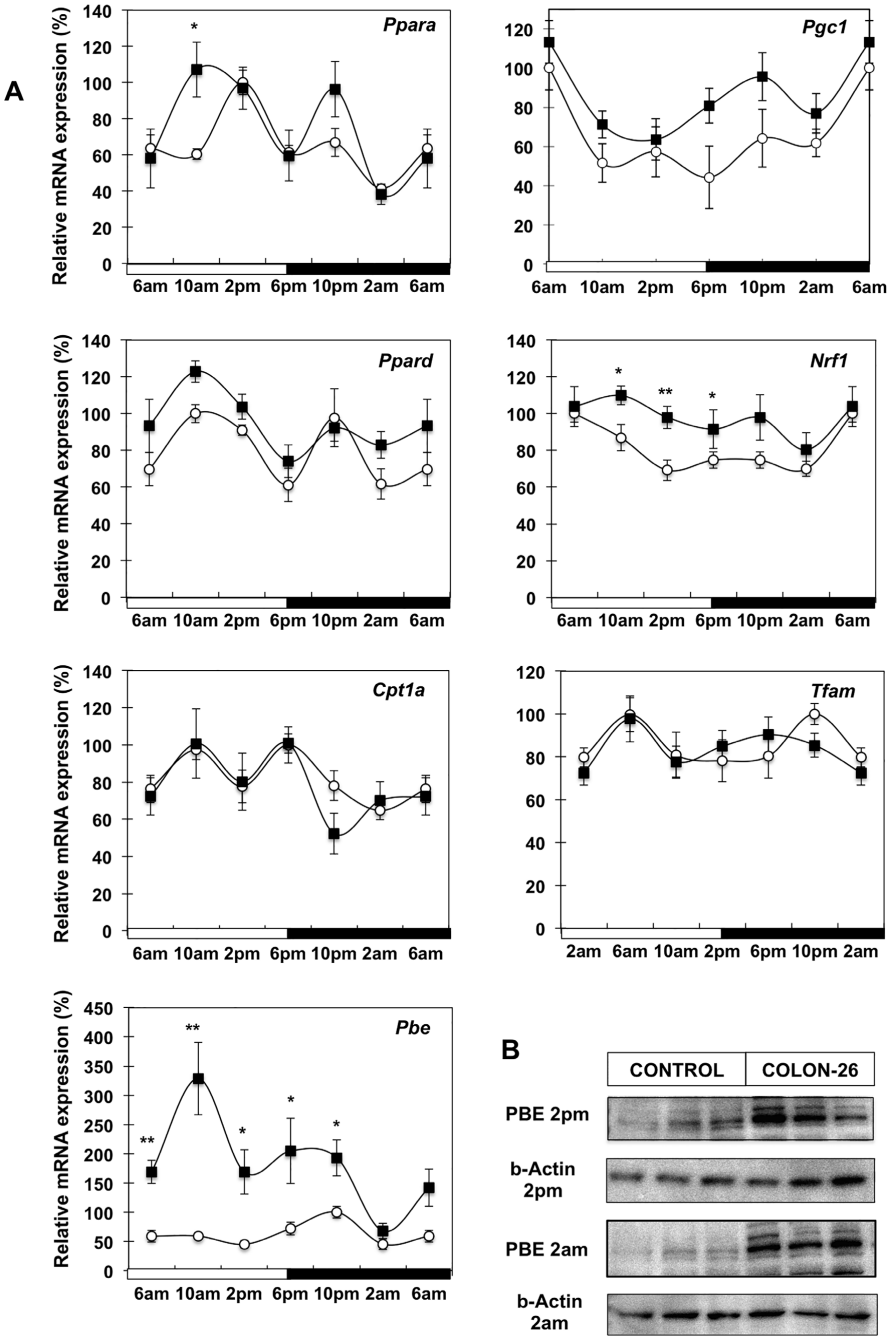
Diurnal expression of genes in lipid utilization pathways in cachectic mice. (A) mRNA analysis of *Pparα, Pgc1α, Pparδ*, *Nrf1, Tfam, Cpt1α* and *Pbe* from WAT of control (white circle) and C26 tumour -bearing mice (black square); Values are mean ± s.e.m. presented as percentages relative to the controls for 4–5 animals per group (*P<0.05; **P<0.01; C26 vs control). 6 am values are duplicated in each graph to illustrate a complete 24-h cycle; (B) Western blot analysis of PBE protein at 2 pm and 2 am time points.

**Figure 6:**
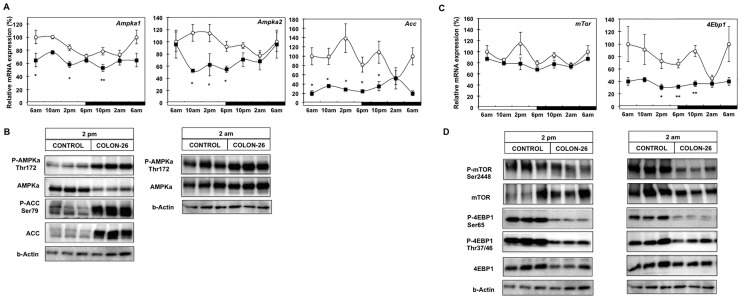
Involvement of AMPK and mTOR/4EBP1 signaling pathways in cancer cachexia. (A) mRNA analysis of *Ampkα1, Ampkα2* and *Acc* from WAT of control (white circle), and C26-bearing mice (black square); (B) Western blot analysis of phospho-AMPK (Thr192) and total AMPK at 2 pm and 2 am; Immunoblot analysis of Phospho-ACC and total ACC for 2 pm WAT samples. (C) mRNA analysis of *mTor* and *4Ebp1;* from WAT of control (white circle), and C26-bearing mice (black square); (D) Western blot analysis of phospho-mTOR (Ser2448) total mTOR, phospho-4EBP1 (Ser65), phospho-4EBP1 (Thr37/46) and total 4EBP1 at 2 pm and 2 am. Values are mean ± s.e.m. presented as percentages relative to the controls for 4–5 animals per group (*P<0.05; **P<0.01; C26 vs control). 6 am values are duplicated in each graph to illustrate a complete 24-h cycle.

An important function of WAT is lipid consumption through the activation of fatty acid β-oxidation in mitochondria and peroxisomes. To understand the role of WAT in the generation of free fatty acids as fuel source during cancer cachexia, we investigated the diurnal expression of transcription factors involved in regulating fatty acid catabolism and corresponding target genes (Figure 5A). In accord with previous reports, *Pparα* exhibited circadian oscillation while *Pparδ* did not show rhythmicity in control mice. There was no significant change apparent for either *Pparα* or *Pparδ* between control and cachectic mice. Similarly *Pgc1α* had a distinctive diurnal pattern of expression, but was unaltered in WAT of cachectic mice. *Nrf1*, a key regulator of metabolic genes involved in mitochondrial respiration, did not show circadian oscillation, however it was significantly increased at certain times during the 24-hour period. Tfam is a master regulator of mitochondrial biogenesis. Its transcript levels did not display rhythmicity and significant changes were not evident during cancer cachexia. Similarly to *Tfam*, expression of carnitine palmitoyl transferase 1α (*Cpt1α)* required for uptake of long chain fatty acids into mitochondria was maintained in C26 tumor-bearing animals. The PPAR target gene, peroxisomal bifunctional enzyme (*Pbe*) involved in peroxisomal fatty acid β-oxidation, exhibits a diurnal peak at 10 pm in the dark cycle in healthy mice. WAT from cachectic mice showed higher abundance of *Pbe* mRNA at most times, as well as a significant 12-hour phase advance, peaking at 10 am (Figure 6A). In addition significant changes were observed between cachectic mice and pair-fed mice at 2 pm (Figure S4). Further evidence of enhanced peroxisomal β-oxidation is the higher protein level of PBE in cachectic animals at both 2 am and 2 pm (Figure 5B, Figure S4). However it appears that other components of fatty acid utilization such as AOX, HADHA and HADHB are unaltered (Figure S5).

### Activation of AMPK Pathway in C26-bearing Animals

AMP-activated protein kinase (AMPK) is a major regulator of energy homeostasis, responding to increased AMP:ATP ratios by stimulating fatty acid oxidation and increasing glucose uptake [32]. One of the primary roles of AMPK is the phosphorylation and consequent inhibition of acetyl CoA carboxylase (ACC), the rate-limiting enzyme in fatty acid synthesis. We investigated mRNA expression levels of the AMPK subunits *Ampkα1* and *α2*, and phosphorylation levels of AMPKα protein as well as expression of ACC (Figure 6A,B, Figure S6). As previously reported [33], we did not observe circadian oscillation with *Ampkα1*, *α2*, while ACC did exhibit cycling with a peak at 2pm in control mice. In cachectic animals reduced mRNA expression for some of the time points during the 24 h period for *Ampkα1* and *2,* and for most time points for *Acc,* was apparent. Similarly to the mRNA expression, AMPK total protein levels also followed the same pattern exhibiting reduced expression in cachectic mice at 2 pm while remaining unaffected at 2 am. The activation of AMPK was confirmed by increased phosphorylation, especially at 2 pm in WAT from C26 tumor-bearing animals. Furthermore, contrary to the mRNA expression we observed increased total protein levels of ACC and significantly increased phosphorylation of ACC at 2 pm in WAT from cachectic animals. These data indicate that inhibition of lipid synthesis during cancer cachexia is associated with activation of AMPK.

### Inhibition of mTOR/4EBP1 Pathway in Cachectic Animals

The mammalian Target of Rapamycin (mTOR) pathway is a master regulator of body mass and energy balance, integrating diverse environmental cues to stimulate anabolic and inhibit catabolic processes via modulation of lipid and protein synthesis. One of the downstream effectors indicative of mTOR action is eukaryotic translation initiation factor 4E binding protein (4Ebp). Therefore, we investigated mRNA expression levels of *mTor* and *4Ebp1* genes, as well as phosphorylation state of mTOR and 4EBP1 proteins (Figure 6C,D, Figure S6). The lack of rhythmic expression of *mTor* transcript levels in control mice was not affected during cancer cachexia. On the other hand *4Ebp1* showed diurnal cycling with a nadir at 2 am and reduced expression at most time points in cachectic mice. Reduced phosphorylation of mTOR was observed at 2 pm and 2 am in WAT samples from C26 tumour-bearing animals indicating inhibition of this regulator during cancer cachexia. Reduced phosphorylation at two separate sites in the 4EBP1 protein was evident in cachectic animals. Preliminary analysis in pair-fed animals also demonstrated a greater reduction in total levels and phosphorylation of mTOR and 4EBP1 proteins (Figure S7). These data indicate that the mTOR/4EBP1 pathway is affected during cachexia, however not as severely as during caloric restriction.

### Expression of Circadian Rhythm Regulators

**Figure 7:**
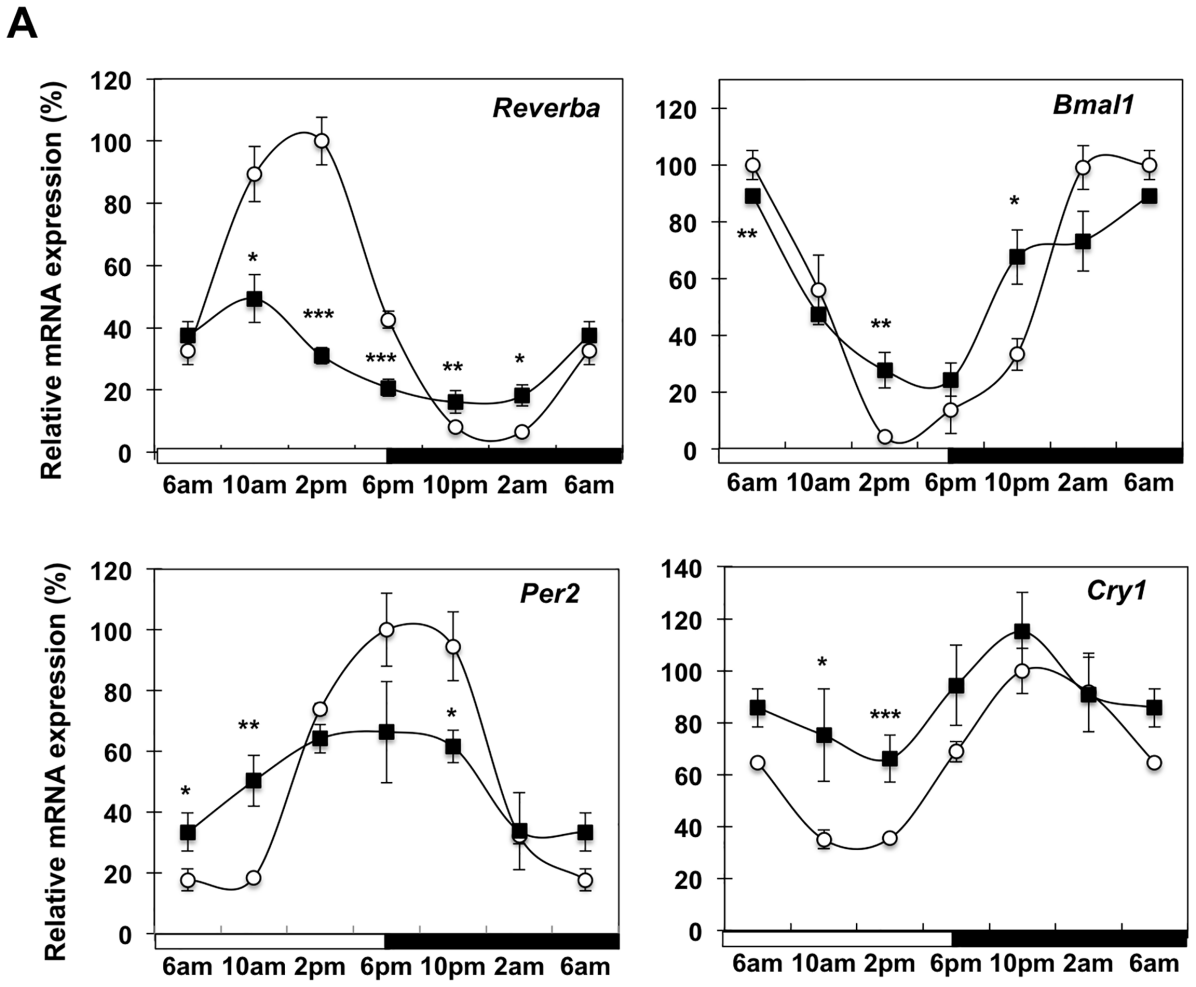
Expression of core clock genes in white adipose tissue during cancer cachexia. mRNA analysis of *Rev-erbα*, *Bmal*, *Per2* and *Cry1* isolated from WAT of control (white circle), and C26-bearing mice (black square); Values are mean ± s.e.m. presented as percentages relative to the controls for 4–5 animals per group (*P<0.05; **P<0.01; ***P<0.001 C26 vs control). 6 am values are duplicated in each graph to illustrate a complete 24-h cycle.

In order to further understand the impact of cancer cachexia on circadian regulation of energy homeostasis in WAT, we investigated the diurnal expression of the major intrinsic clock oscillator genes *Rev-erbα*, *Bmal*, *Per2* and *Cry1*. Similarly to Yang et al [18], all four genes exhibited a strong diurnal expression in WAT of control mice (Figure 7). Cachexia resulted in loss of rhythmicity of *Rev-erbα* and *Per2*, with distinct increases and reductions at specific time-points diminishing the net diurnal amplitude between the peaks and troughs of expression. *Bmal1* and *Cry1* mRNA levels were also altered with increased expression observed at the nadir of expression.

## Discussion

One of the most prominent symptoms of cancer cachexia is the involuntary loss of weight due to fat depletion and muscle wasting that cannot be accounted for by reduced food intake. Both clinical and experimental animal studies involving tumour-bearing mouse models of cachexia, indicate that accelerated lipid depletion is responsible for the loss of adipose tissue [25], [26], [34], [35]. In addition, recent work performed in our laboratory demonstrated that lipid metabolic and thermogenic pathways are switched on in brown adipose tissue (BAT) during cancer cachexia and coincide with elevated BAT temperatures at specific times of the diurnal cycle [8]. These observations prompted us to elucidate the impact of cancer cachexia on white adipose tissue with a specific focus on the control of circadian rhythm, the diurnal expression of key lipid metabolic regulators and homeostatic signaling pathways such as the AMPK and mTOR/4EBP1 in the context of active cytokine signaling.

In agreement with previous reports, we observed diminished WAT mass accompanied by a reduction in adipocyte size in C26 tumour-bearing mice [30], [34], [36]. Loss of fat occurred alongside increased circulating free fatty acids and decreased triglycerides in plasma of cachectic mice. The morphological changes in adipocytes appear to be more severe in cachectic compared to pair-fed animals, indicating that reduced food intake alone is not sufficient to account for the extent of fat depletion in cancer cachexia.

Many factors have been implicated in cancer cachexia including cytokines (TNFα, IL-6), ZAG and myostatin [36], [37]. The C26 model of cachexia is characterized by elevated plasma levels of IL-6. We document increased phosphorylation of STAT3 protein together with elevated transcript levels of the STAT3 target gene - *Socs3* (Figure 2). In combination, these findings provide evidence for active cytokine signaling in WAT of cachectic mice, consistent with the suggestion that IL-6 and STAT3 contribute significantly to the muscle wasting [14], activation of thermogenesis in BAT [8] and acute phase protein production in the C26-model of cancer cachexia [38]. Earlier studies show IL-6 administration in mice and humans enhances lipolysis and fat oxidation, while *in vitro*, IL-6 can directly stimulate lipolysis in adipocyte cell culture [33]. The development of obesity in IL-6 knockout mice further reinforces the key role of this cytokine in controlling lipid metabolism [11], [39]. Therefore, it is likely that the same signaling pathway drives the fat depletion in WAT during cancer cachexia.

A close relationship between circadian rhythm and regulation of metabolism in white adipocytes exists, with up to 20% of the murine and 25% of the human adipose tissue transcriptome exhibiting diurnal oscillation [17], [40]. We investigated expression of various transcription factors, respective target genes involved in lipid metabolism as well as core regulators of the circadian clock machinery in cachectic animals. We observed reduced amplitudes in the mRNA expression of *Reverbα Per2, Pparγ C/ebpα*, and associated genes (*Fas, Lpl, Scd1, Dgat2*) and a parallel increase in *Bmal1, Cry1, Pbe* and *Tfam* indicating alterations in the core clock regulation and lipid homeostasis during cachexia. BMAL and REVERB proteins have been implicated in the regulation of adipogenesis [22]. Perturbations to the circadian clock machinery may be responsible for the alterations in lipid metabolic pathways resulting in WAT depletion. However the relative contributions of anorexia versus direct cytokine signaling within WAT to the diurnal perturbations apparent in C26 cachectic WAT is unclear at this stage.

Consistent with our observations, other animal models of cancer cachexia exhibit impaired lipogenesis through reduced PPARγ action during cancer cachexia [34], [41]. However evidence from the limited number of clinical studies currently available do not support a major role for inhibition of lipogenesis during the development of cachexia in cancer patients [25], [26].

On the other hand, fat depletion is likely accelerated through triglyceride degradation and fatty acid β-oxidation as indicated by increased ATGL and PBE expression (Figure 5 and 6). While other studies have linked fat tissue depletion to lipid catabolism during cachexia [25], [26], [34], we demonstrate for the first time increased protein levels of ATGL and PBE at two time points representing the mid-points of the day and night cycle. This observation is consistent with the increased lipase activities evident in WAT of cachectic cancer patients as well as tumour-bearing mice [42], [43]. The increased lipid hydrolase activity in WAT of C26 mice appears to be mainly attributable to ATGL rather than HSL (Figure 1). Interestingly, lipid catabolism does not appear to be stimulated through PKA/HSL phosphorylation and activation but rather through increased protein levels of ATGL (Figure 4). While there are many confounding factors, such as post-translational repression and association with lipid droplets, in relating HSL and ATGL activities to their respective mRNA and protein abundance, the marked decrease in HSL gene expression suggests an active signal to repress HSL in contrast to the apparently enhanced ATGL protein levels.

Our findings with the C26 model of cachexia are consistent with previous work showing that ATGL knockout animals are more resistant to lipolysis than HSL deficient mice when inoculated with B16 melanoma or Lewis lung carcinoma [24]. The same study also reported loss of muscle mass was prevented in absence of ATGL, further reinforcing the integral role of this lipase in cancer cachexia syndrome [42], [43]. Similarly to ATGL, we observed increased levels of perilipin protein in WAT of cachectic mice (Figure 4). Assuming the phosphorylation status of this protein remains unchanged, this finding might indicate a more limited access to the remaining triglyceride pool at late stage cachexia. However this observation could also be due to the presence of smaller lipid droplets and subsequently increased area of lipid monolayers (and thus perilipin content). Recent studies in rats have shown that during prolonged fasting ATGL and HSL are not coordinately regulated. During phase 3 of fasting when protein catabolism predominates, ATGL protein levels increase, while HSL phosphorylation levels decrease. Therefore, our observations indicate that during late stage cancer cachexia the metabolic status of WAT may have certain features in common with the last phase of fasting [44]. This notion is also supported by the presence of active PKA/HSL in pair-fed animals, which is more consistent with phase 2 fasting when lipid mobilization is predominant. The findings from the current study demonstrate that during cancer cachexia lipid mobilization is enhanced in a manner resembling a prolonged fasting state that may potentially accelerate protein degradation. AMPK is an important sensor of nutrient levels in the cell. Its activation has been associated with stimulation of fatty acid oxidation and glucose uptake [45]. Furthermore AMPK is an important component of ATGL regulation as it directly phosphorylates ATGL to increase hydrolase activity [46]. Our study shows enhanced phosphorylation of AMPK in WAT of cachectic mice as well as its target ACC. Phosphorylation of ACC would be manifested as inhibition of fatty acid synthesis concomitant with increased mitochondrial fatty acid oxidation [47]. In combination with the increased levels of ATGL protein (Figure 4), activation of AMPK during cachexia (Figure 6) could result in enhanced triglyceride lipolysis mediated by ATGL. There is increasing evidence for links between cytokines such as IL-6 and AMPK. For example IL-6 knockout mice exhibit decreased AMPK activity in muscle and adipose tissue [48]. It is possible that cytokines such as IL-6 enhance AMPK activation and through this mechanism, stimulate lipid mobilization from WAT during cancer cachexia. Another downstream effector of AMPK is the mTOR/4EBP1signaling pathway that controls a plethora of cellular functions including protein synthesis, lipid metabolism and cell growth in response to nutritional state [49]. Our study demonstrated inhibition of the mTOR/4EBP1 pathway through reduced phosphorylation of both proteins. Transcriptomic analysis of WAT from cachectic cancer patients found down-regulation of several pathways, including extracellular matrix, cytoskeleton and cellular adhesion was significantly associated with fat loss during cachexia [9]. As these cellular structures represent a significant component of the protein content of adipocytes, our finding of diminished mTOR-related signaling could provide a mechanism for how adipocytes reduce protein synthesis to adapt their cell volume as they deplete lipid reserves and shrink during cachexia. Changes in caloric intake are known to directly negatively influence translational initiation factors and ribosomal proteins to reduce energy expenditure on protein production [50]. It appears that in the current study of WAT, cancer cachexia shares some similarities with caloric restriction. As such further studies are required to compare cancer cachexia and caloric restriction in order to understand how protein synthesis and associated lipid homeostasis pathways through AMPK/mTOR and 4EBP1 are affected.

Apart from PBE, there does not appear to be a generalized increase in other components of either peroxisomal or mitchondrial fatty acid β-oxidation coordinated by PPARα, PPARδ or PGC1α in WAT of cachectic mice (Figure 5; Figure S2). Therefore it is possible that lipid mobilization from WAT supplies substrates for fatty acid utilization in other organs. In this regard, our recent demonstration of activated thermogenesis in BAT of C26 cachectic mice [8] and increased fatty acid oxidation rates [30] may indicate the potential fate for a significant amount of fatty acids catabolized from WAT. Such energetically wasteful utilization of lipids for heat production does not occur in BAT of pair-fed mice [8] that are also undergoing rapid lipolysis of their fat stores in WAT.

In conclusion, here we demonstrate disrupted circadian expression of clock regulators and key genes involved in lipid homeostasis in WAT of cachectic mice. Reduced fat accumulation, combined with increased fat mobilization and fatty acid oxidation is associated with increased cytokine signaling through STAT3 and AMPK. Triglyceride breakdown does not appear to be stimulated by the classic lipolytic pathway mediated by PKA stimulation of hormone sensitive lipase, but rather through ATGL. Furthermore there is reduced mTOR signaling in parallel with altered lipid metabolism in WAT of cachectic mice. Finally, our data indicate that these signaling pathways are affected at two times at opposite points of the diurnal cycle indicating perturbed temporal regulation of adipose energy homeostasis. Improved understanding of these alterations in lipid homeostasis in cancer cachexia may assist clinicians to manage this metabolic disorder in advanced cancer patients.

## Supporting Information

Figure S1 Circadian expression pattern of lipid uptake in cachectic mice. mRNA analysis of *Cd36* and *Ap2* isolated from WAT of control (white circle), and C26-bearing mice (black square); Values are mean ± s.e.m. presented as percentages relative to the controls for four-five animals per group (*P<0.05; **P<0.01; ***P<0.001 C26 vs control). 6 am values are duplicated in each graph to illustrate a complete 24-h cycle. mRNA analysis of *Cebpα, Fas,* isolated from WAT of control (white circle), and C26-bearing mice (black square) and Pair-fed mice (grey square); Values are mean ± s.e.m. presented as percentages relative to the controls for four-five animals per group (2 am values are duplicated in each graph to illustrate rhythmicity); (***P<0.001 C26 vs control, $ P<0.05 PF vs control, $$ P<0.01 PF vs control, # P<0.05 C26 vs PF, ## P<0.01 C26 vs PF, ### P<0.001 C26 vs PF).
(TIF)

Figure S2 Densitometric analysis of bands. Relative values for phosphorylated and total HSL, PKA, and for total protein levels of Perilipin and ATGL were determined by densitometric analysis and were normalized against beta-actin. Values are mean ± s.e.m. presented as percentages relative to the controls (*P<0.05; **P<0.01; ***P<0.001 C26 vs control).
(TIFF)

Figure S3 PKA-mediated lipolysis pathway in WAT of control, cachectic and pair-fed animals. Western blot analysis of phospho-PKA (Thr197) total PKA, phospho-HSL and total HSL, ATGL and Perilipin at 2 pm. Relative values for phosphorylated and total HSL, PKA, and for total protein levels of Perilipin and ATGL were determined by densitometric analysis and were normalized against beta-actin. Values are mean ± s.e.m. presented as percentages relative to the control mice (*P<0.05 C26 vs control, **P<0.01 C26 vs control, $ P<0.05 PF vs control, $$ P<0.01 PF vs control, $$$ P<0.001 PF vs control, # P<0.05 C26 vs PF, ## P<0.01 C26 vs PF). mRNA analysis of *Hsl* and isolated from WAT of control (white circle), and C26-bearing mice (black square) and Pair-fed mice (grey square); Values are mean ± s.e.m. presented as percentages relative to the controls for four-five animals per group (2 am values are duplicated in each graph to illustrate rhythmicity); (**P<0.01 C26 vs control, ***P<0.001 C26 vs control, ### P<0.001 C26 vs PF).
(TIF)

Figure S4 Gene expression comparison between 3 groups and densitometric analysis of PBE protein bands. mRNA analysis of *Pbe* isolated from WAT of control (white circle), and C26-bearing mice (black square) and Pair-fed mice (grey square); Values are mean ± s.e.m. presented as percentages relative to the controls for four-five animals per group (2 am values are duplicated in each graph to illustrate rhythmicity); (**P<0.01 C26 vs control, ## P<0.01 C26 vs PF). Relative values for total protein levels of PBE were determined by densitometric analysis and were normalized against beta-actin. Values are mean ± s.e.m. presented as percentages relative to the controls (*P<0.05; **P<0.01; ***P<0.001 C26 vs control).
(TIF)

Figure S5 Circadian expression pattern of beta-oxidation in cachectic mice. mRNA analysis of *Aox, Hadha* and *Hadhb* isolated from WAT of control (white circle), and C26-bearing mice (black square); Values are mean ± s.e.m. presented as percentages relative to the controls for four-five animals per group (6 am values are duplicated in each graph to illustrate a complete 24-h cycle.
(TIFF)

Figure S6 Densitometric analysis of bands. Relative values for phosphorylated and total AMPK, ACC, mTOR and 4EBP1 were determined by densitometric analysis and were normalized against beta-actin. Values are mean ± s.e.m. presented as percentages relative to the controls (*P<0.05; **P<0.01; ***P<0.001 C26 vs control).
(TIFF)

Figure S7 mTOR/4EBP1 pathway in WAT of control, cachectic and pair-fed animals. Western blot analysis of phospho-mTOR (Ser2448), total mTOR phospho-4EBP1 (Ser65, Thr46/37) and total 4EBP1. Relative values for phosphorylated and total mTOR and 4EBP1 were determined by densitometric analysis and were normalized against beta-actin. Values are mean ± s.e.m. presented as percentages relative to the control mice (*P<0.05 C26 vs control, ***P<0.001 C26 vs control, $$$ P<0.001 PF vs control, # P<0.05 C26 vs PF).
(TIF)

Table S1 qPRR primer list.
(DOCX)
