# Supplementary figures and images for: Depletion of White Adipose Tissue in Cancer Cachexia Syndrome Is Associated with Inflammatory Signaling and Disrupted Circadian Regulation
Source: PLoS One. 2014 Mar 25;9(3):e92966. doi: 10.1371/journal.pone.0092966 (PMC3965507; doi:10.1371/journal.pone.0092966)

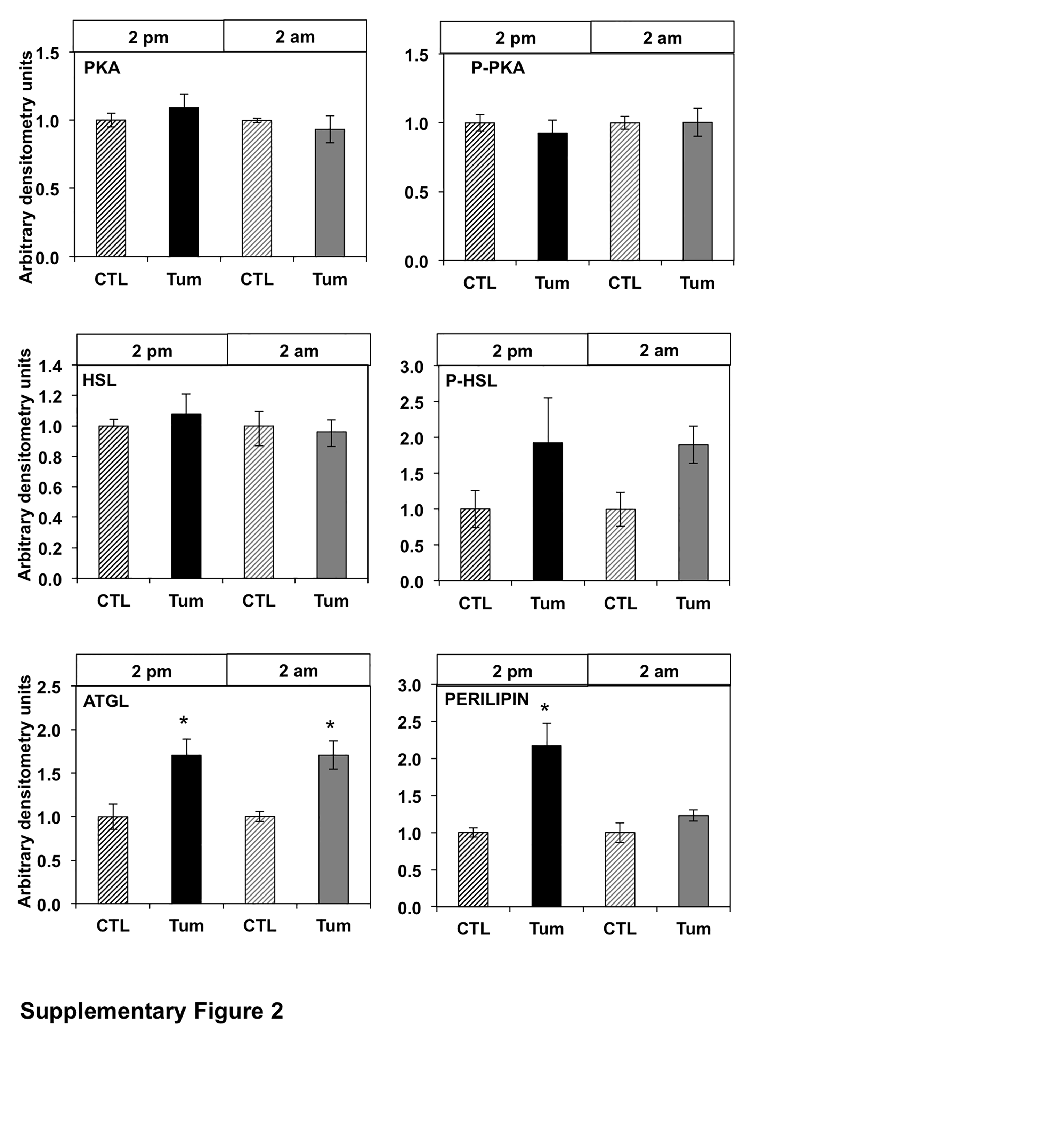

Supplement: Figure S2 — Densitometric analysis of bands. Relative values for phosphorylated and total HSL, PKA, and for total protein levels of Perilipin and ATGL were determined by densitometric analysis and were normalized against beta-actin. Values are mean ± s.e.m. presented as percentages relative to the controls (*P<0.05; **P<0.01; ***P<0.001 C26 vs control). (TIFF) [file pone.0092966.s002.tiff]

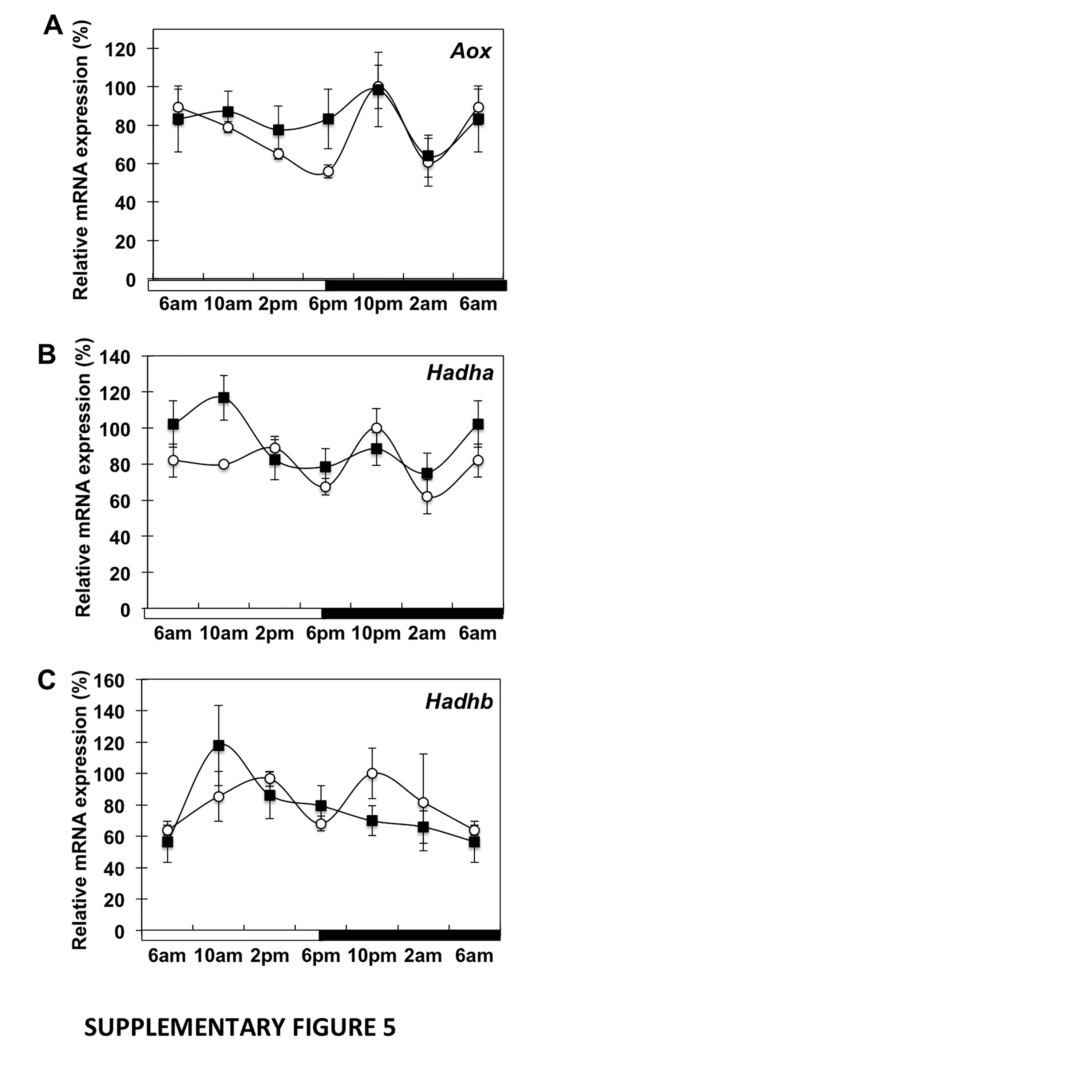

Supplement: Figure S5 — Circadian expression pattern of beta-oxidation in cachectic mice. mRNA analysis of Aox, Hadha and Hadhb isolated from WAT of control (white circle), and C26-bearing mice (black square); Values are mean ± s.e.m. presented as percentages relative to the controls for four-five animals per group (6 am values are duplicated in each graph to illustrate a complete 24-h cycle. (TIFF) [file pone.0092966.s005.tiff]

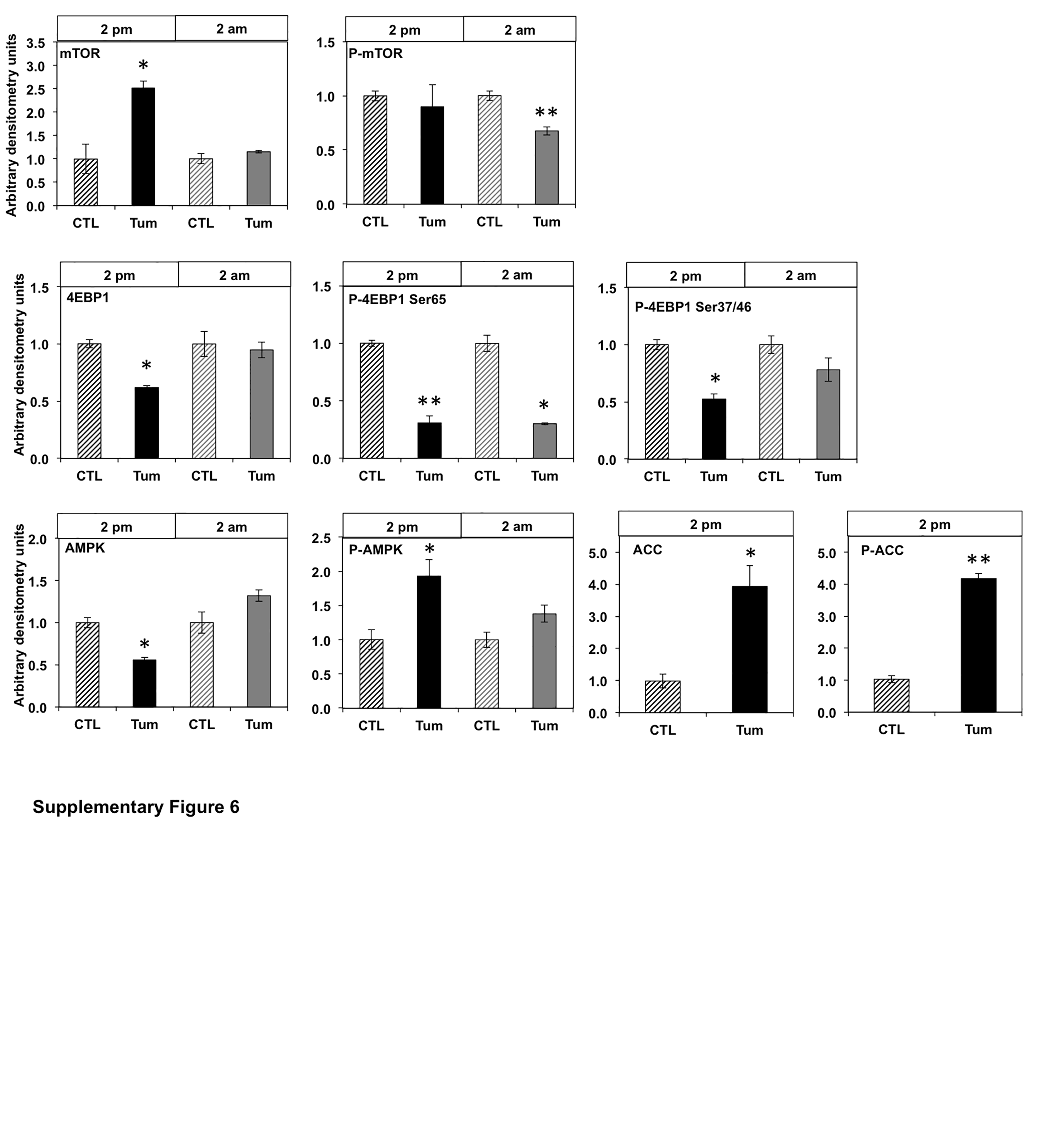

Supplement: Figure S6 — Densitometric analysis of bands. Relative values for phosphorylated and total AMPK, ACC, mTOR and 4EBP1 were determined by densitometric analysis and were normalized against beta-actin. Values are mean ± s.e.m. presented as percentages relative to the controls (*P<0.05; **P<0.01; ***P<0.001 C26 vs control). (TIFF) [file pone.0092966.s006.tiff]

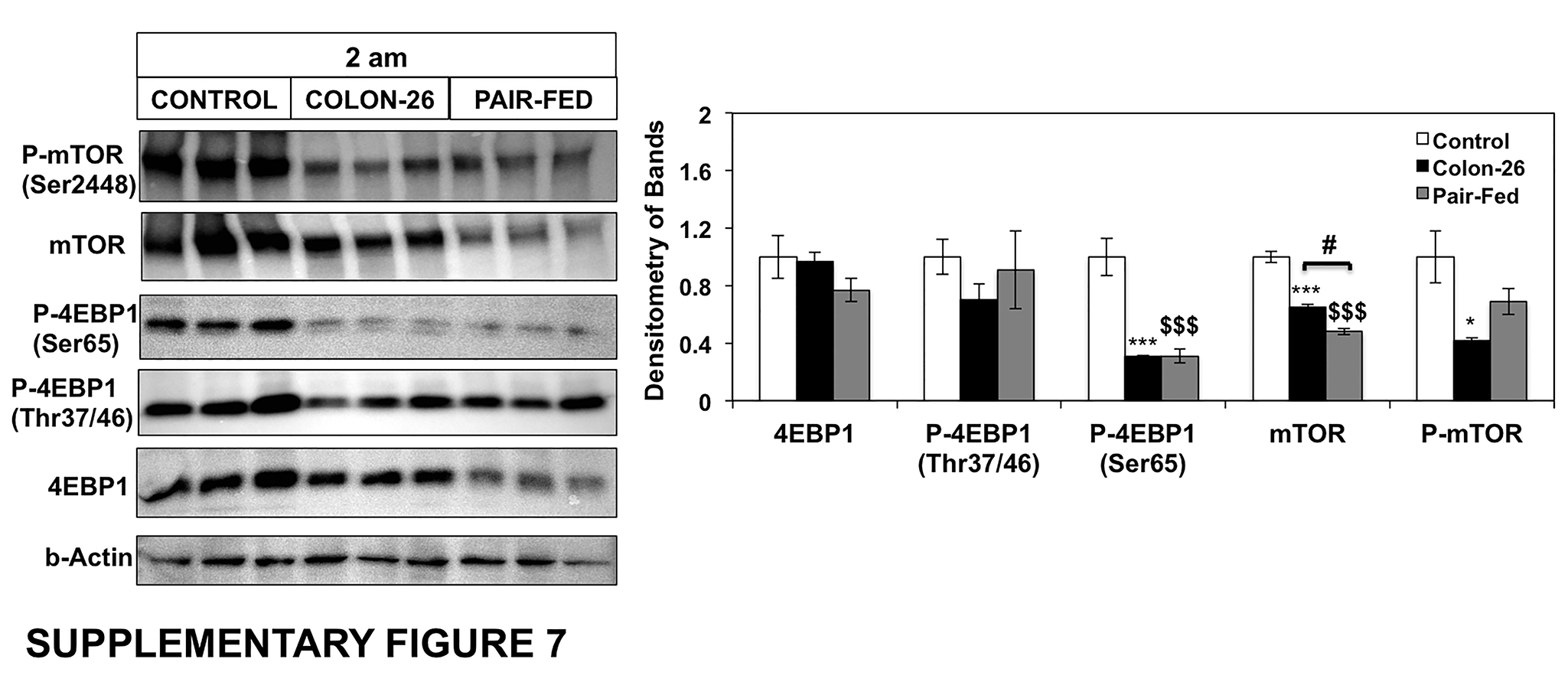

Supplement: Figure S7 — mTOR/4EBP1 pathway in WAT of control, cachectic and pair-fed animals. Western blot analysis of phospho-mTOR (Ser2448), total mTOR phospho-4EBP1 (Ser65, Thr46/37) and total 4EBP1. Relative values for phosphorylated and total mTOR and 4EBP1 were determined by densitometric analysis and were normalized against beta-actin. Values are mean ± s.e.m. presented as percentages relative to the control mice (*P<0.05 C26 vs control, ***P<0.001 C26 vs control, $$$ P<0.001 PF vs control, # P<0.05 C26 vs PF). (TIF) [file pone.0092966.s007.tif]
