# Supplementary material for: Depletion of White Adipose Tissue in Cancer Cachexia Syndrome Is Associated with Inflammatory Signaling and Disrupted Circadian Regulation
Source: PLoS One. 2014 Mar 25;9(3):e92966. doi: 10.1371/journal.pone.0092966 (PMC3965507; doi:10.1371/journal.pone.0092966)
Supplement: Table S1 — qPRR primer list. (DOCX) [file pone.0092966.s008.docx]

Table S1: qPRR primer list

| **Gene** | **Accession Number** |  | **Sequence** |
| --- | --- | --- | --- |
| **36B4** | NM_007475 | F | AGATGCAGCAGATCCGCATGTC |
|  |  | R | AGCAGCAGCTGGCACCTTATTG |
| **BMAL1** | NM_007489 | F | CCAAGAAAGTATGGACACAGA |
|  |  | R | GCATTCTTGATCCTTCCTTGG |
| **CD36** | NM_007643 | F | TAATGGCACAGACGCAGCCTC |
|  |  | R | GTGATGCAAAGGCATTGGCTGG |
| **CEBPA** | NM_007678 | F | AGAGCCGAGATAAAGCCAAACA |
|  |  | R | CGGTCATTGTCACTGGTCAACT |
| **CLOCK** | NM_007715 | F | GTGAGTCTCAAGGAAGCACTGGA |
|  |  | R | GACCAACTGACTGGGAGTTTATGG |
| **CPT1A** | NM_013495 | F | CCTGCCTGTCCCAGCTGTCAA |
|  |  | R | GTGCTGTCATGCGTTGGAGTC |
| **CRY1** | NM_007771 | F | GGAGATGCCAGCAGACACCATC |
|  |  | R | CGTTCAAAGTTTGCCACCCAGGC |
| **DGAT2** | NM_026384 | F | GGCTACGTTGGCTGGTAACTTC |
|  |  | R | TTGCCAGGCATGGAGCTCAG |
| **FAS** | NM_007988 | F | CCTGGATAGCATTCCGAACCT |
|  |  | R | AGCACATCTCGAAGGCTACAC |
| **GAPDH** | NM_008084 | F | GTCGTGGATCTGACGTGCC |
|  |  | R | TGCCTGCTTCACCACCTTCT |
| **HMBS** | NM_013551 | F | TTGGAAAGACCCTGGAAACCTTG |
|  |  | R | GACCCACAGCATACATGCATTCT |
| **HSL** | NM_010719 | F | GCT GGG CTG TCA AGC ACT GT |
|  |  | R | GTA ACT GGG TAG GCT GCC AT |
| **PBE** | NM_023737 | F | GCTGGCCTTGGGCTGTCAC |
|  |  | R | GCGTTCCTCTTGCACCAGGAAC |
| **PER2** | NM_011066 | F | TGCTTGTTCCAGGCTGTGGATGA |
|  |  | R | ACCCTGACTTTGTGCCTCCCAA |
| **PERILIPIN** | NM_175640 | F | GGCCTGGACGACAAAACC |
|  |  | R | CAGGATGGGCTCCATGAC |
| **PGC1α** | NM_008904 | F | AGCCGTGACCACTGACAAGGA |
|  |  | R | GCTGCATGGTTCTGAGTGCTA |
| **PPARα** | NM_011144 | F | ACAAGGCCTCAGGGTACCA |
|  |  | R | GCCGAAAGAAGCCCTTACAG |
| **PPARγ** | NM_011146 | F | TTGACCCAGAGCATGGTGC |
|  |  | R | GAAGTTGGTGGGCCAGAATG |
| **PPARδ** | NM_011145 | F | GCCTCGGGCTTCCACTAC |
|  |  | R | AGATCCGATCGCACTTCTCA |
| **REV-ERBα** | NM_145434 | F | GGGCACAAGCAACATTACCA |
|  |  | R | CACGTCCCCACACACCTTAC |
| **SCD1** | NM_009127 | F | TCACGACCCCACCTATCAGG |
|  |  | R | TTCCTCCAGACGTACTCCAGC |
| **SOCS3** | NM_007707 | F | GGCCACCCTCCAGCATCTTTG |
|  |  | R | GTGGCAGCTCCCCCTCCCCTC |
|  |  |  |  |
|  |  |  |  |
|  |  |  |  |
